# Agricultural Economic Risk Forecast Based on Data Mining Technology

**DOI:** 10.1155/2022/3684736

**Published:** 2022-04-27

**Authors:** Lei Wang, Hongwei Tan

**Affiliations:** ^1^Economics and Management School, Jilin Agricultural University, Changchun 130118, China; ^2^Business School, Changchun Sci-Tech University, Changchun 130600, China; ^3^Language and Culture School, Changchun Sci-Tech University, Changchun 130600, China

## Abstract

In order to improve the effect of agricultural economic risk forecast, this paper studies the agricultural economic risk forecast combined with data mining technology and builds an intelligent agricultural economic risk forecast system. Moreover, this paper employs a dynamic factor model to estimate common factors that drive changes in target topics. In order to construct a sentiment index that can reflect the overall operating situation of the macroeconomy, this paper improves the agricultural economic risk mining algorithm and standardizes the sentiment value corresponding to the target theme. In addition, this article analyzes the sentiment changes of its individual topics one by one in combination with the specific economic environment. The simulation study shows that the agricultural economic risk forecast system based on data mining technology proposed in this paper has a good effect.

## 1. Introduction

Market risk refers to the uncertainty of whether agricultural producers and operators can successfully sell agricultural products produced by using agricultural scientific and technological achievements or whether individual labor can be converted into social labor for profit in agricultural promotion. This is the primary and biggest risk of agricultural extension work under market economy conditions. In the process of agricultural marketization, the important basis for agricultural extension is market information, including market supply and demand information and price information.

Thousands of scattered market entities are induced by market prices and interests, lack of information connection with each other, and are excluded or lack cooperation due to competition and eventually fall into the market “trap.” As a result, the “thrilling jump” failed, which not only broke the commodity but also feared the producer itself. Its actual performance is structural convergence, waste of resources, and damage to farmers [1]. Since agriculture takes living organisms as the production and operation object, the products are destructible and intolerant of storage [2]. At the same time, the planting industry in agriculture has a wide spatial distribution and a long production cycle and is greatly constrained by the natural environment and natural conditions. Various natural disasters in nature, such as floods, droughts, hail, frost, rain and little sunshine, low temperatures, and pests and diseases, will cause losses to agricultural production. In light cases, it will lead to the reduction of production and income, and in heavy cases, there will be no gains, thus forming natural risks of agricultural production and agricultural extension [3].

Generally speaking, agricultural producers and operators will be induced by market information and simply follow the principle of “whatever is in short supply, whatever is high in price, and what is more profitable, just plant or raise whatever” to implement and apply new agricultural technologies. Although the market can allocate resources effectively, there are also “traps.” This is because, on the one hand, the information reflected by the market is real-time information, and this information only reflects the nature of the market and cannot reflect the stipulation of market volume;, that is, no matter how big is the gap between supply and demand in the market and how much will be supplied in the market in the future, it cannot be reflected, so that when the supply exceeds the demand, the producers rush up and when the supply exceeds the demand, the producers rush down. On the other hand, the market regulation of agricultural products with a long production cycle has a particularity. According to the “cobweb theory,” the demand for agricultural products is a function of the current price, while the supply of agricultural products is a function of the price of the previous period, that is, the high demand caused by the current supply exceeds the demand. The price induces more supply in the next period, which leads to a decrease in price. The low price caused by oversupply induces a small supply in the next period, and the low supply leads to an increase in price, and the cycle continues. In this way, the production decisions of agricultural product producers are always made based on the price information of the previous period, which eventually leads to periodic “difficulty in selling” agricultural products.

In order to improve the ability to cope with agricultural economic risks, this paper combines data mining technology to study agricultural economic risk forecast and builds an intelligent agricultural economic risk forecast system to improve the effect of agricultural economic risk forecast.

## 2. Related Work

Looking at the history and current situation of agricultural science and technology promotion in various countries in the world, although the political systems, economic levels, agricultural equipment, technical conditions, and the cultural and technical quality of farmers vary from country to country, the agricultural extension systems of most agriculturally developed countries have been privatized and the characteristics of customization, diversification, and integration, agricultural economics, business management, planning, marketing, and other economic sciences, rural sociology, psychology, organization, education, etc., were introduced into the construction of agricultural extension systems. Behavioural sciences such as communication science make the agricultural extension system step into a virtuous circle [4]. Agricultural extension is full of dynamism, showing a vigorous momentum. Most countries are government-run agencies based on the government's Ministry of Agriculture which are the main form of agricultural extension organizations. Even though the United States and the United Kingdom are economically developed and rich in resources, their grain and other major agricultural products are produced in large quantities, but they have not given up on the government. The agricultural extension organization is established as the main body [5]. This fully reflects the importance these countries attach to agricultural extension. In recent years, the proportion of government investment in agricultural extension has tended to decrease. However, this is also due to the gradual improvement of the agricultural extension system in these countries. After entering a virtuous circle, various parties raised funds, the reason for the smoother channels [6]. At the same time, the agricultural extension organizations in most foreign countries implement vertical leadership and comprehensive setting, which avoids a lot of waste of resources caused by the double leadership of “stripes” and “blocks” and repeated institutional settings [7]. In terms of operation mechanism, agricultural extension organizations in most foreign countries are closely linked with agricultural and scientific research and education, forming an operating mechanism of smooth information, exchange of needs, and full cooperation, effectively solving the combination of agricultural extension, scientific research, and educational questions [8].

The study in [9] pointed out that although the operation of “farm yield insurance” for independent and scattered individual farmers is more complicated, the management cost is higher and it faces adverse selection and moral hazard problems, but compared with the government's disaster relief plan, agricultural insurance is a more stable farm income. The study in [10] pointed out that agricultural insurance effectively guarantees the continuity of agricultural production, which is more effective than simply providing financial support. The study in [11] believed that agricultural insurance plays a significant role in promoting agricultural development, reducing poverty, and restoring agricultural production. The study in [12] pointed out the necessity of crop insurance as an antirisk tool. The study in [13] proposed that agricultural insurance is an important part of rural finance, and it is an effective way to diversify risks, which can effectively reduce the risk of farmers using new technologies for production. The study in [14] showed that the development of agricultural insurance has promoted the innovation of agricultural production technology and the stability of income. The study in [15] studied that agricultural economic growth benefits from the development of agricultural insurance to a certain extent, and within a certain range, by increasing agricultural insurance premium income and expanding agricultural insurance claims, it can effectively and positively promote the growth of the agricultural economy. The study in [16] analyzed the development process of agricultural insurance in developed countries and believed that the development of agricultural insurance is a necessary condition for creating modern agriculture. Agricultural insurance has huge development potential and is an indispensable part of modern agricultural economic growth. The study in [17] believed that various combinations of insurance products promote the good development of urban agriculture, and agricultural insurance in the product combination is an indispensable part, which plays a good role in promoting the growth of the agricultural economy [18].

The study in [19] believed that agricultural insurance is conducive to stabilizing the national economy and social life at the macrolevel; at the microlevel, it can compensate farmers' losses, stabilize the source of funds for farmers, and enhance farmers' ability to repay loans. In turn, the economic level will also affect agricultural insurance. On the one hand, the level of economic development affects the supply and demand of agricultural insurance, and on the other hand, the economic policies formulated by the state will also affect the development of agricultural insurance. The study in [20] believed that agricultural insurance can help agricultural production to diversify risks and compensate for losses, thereby helping farmers to stabilize production and increase income. The welfare effects, credit support effects, and policy effects of agricultural insurance will bring favorable multipliers to the entire economy effect. The study in [21] believed that agricultural insurance can achieve capital magnification through various ways, such as the demonstration and assistance role of financial input, the linkage mechanism between banks and credit, and the improvement of the use efficiency of financial input. The study in[22] analyzed the interaction basis and mechanism between agricultural insurance and rural credit and believed that agricultural insurance can make use of its risk diversification function to achieve Pareto improvement of resource allocation in rural credit, thereby increasing agricultural production and developing rural areas.

## 3. Agricultural Economic Data Processing Based on Data Mining

**Figure 1:**
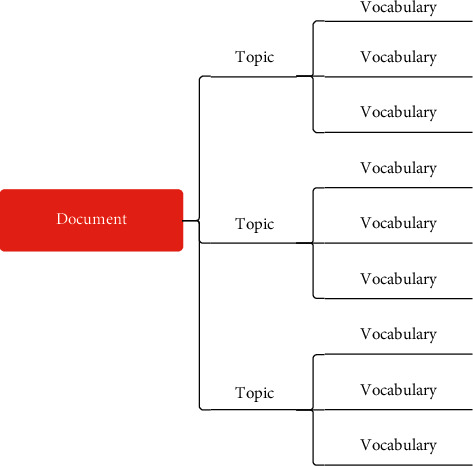
Document structure diagram.

In the process of processing agricultural economic data, the first use of this paper is the latent Dirichlet allocation model, referred to as the LDA model. This model is a document generation model that can be used to identify hidden topic information in large-scale corpora. The model believes that any document in the document set is composed of multiple topics, and each topic is composed of multiple keywords. First, it selects a topic with a certain probability and then selects a certain keyword under the topic with a certain probability. In this way, the first keyword of the document is generated, and the whole document is generated by repeating it continuously. Specifically, a document contains three layers, namely, the document layer, the topic layer, and the vocabulary layer. The document structure diagram is shown in Figure 1.

**Figure 2:**
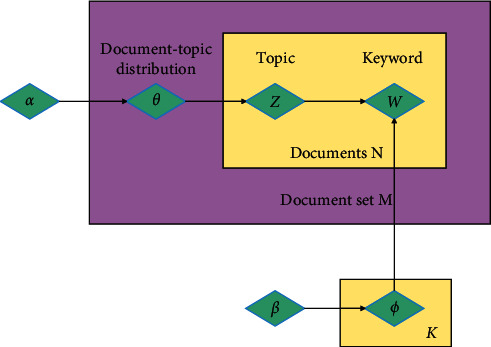
Theoretical structure of the LDA model.

The model assumes that any document is a mixture of multiple topics, the document is the probability distribution of multiple topics, and any topic is the probability distribution of multiple words. On this basis, the model is improved and the Dirichlet distribution prior of topic-word distribution is added. The model representation is shown in Figure 2.

Figure 2 includes hyperparameters *α* and *β*, topic-word distribution, and document-topic distribution. *W* marked in black indicates that the keyword it represents is the only observable variable in the document, and the conditional probability is shown in the direction of the arrow in the figure. The specific steps for generating documents in the LDA model are as follows:

- For any document *d* ∈ *D*, the multinomial distribution parameters *W*_*d*,*j*_ of the topics on document *d* can be obtained from *θ*_*d*_ ~ *Dir*(*α*)
- For any topic *z* ∈ *K*, the multinomial distribution parameter *ϕ*_*z*_ of the vocabulary on topic *z* can be obtained from *j*_*z*_ ~ *Dir*(*β*)
- It selects the vocabulary *W*_*d*,*j*_ in the document *d*, the topic *z*_*d*,*j*_ can be obtained from the multinomial distribution *z*_*d*,*j*_ ~ *Mult*(*θ*_*d*_), and the vocabulary *W*_*d*,*j*_ is obtained according to the multinomial distribution *W*_*d*,*j*_ ~ *Mult*(*θ*_*d*_)

In LDA models, *θ* and *φ* cannot be obtained directly; therefore, the two parameter values are usually approximated by parameter estimation methods.

In order to identify the covariability characteristics of the economic cycle, this paper uses the dynamic factor model method to estimate the selected seven representative target themes and obtains the common factors that drive their changes. For a long time, the dynamic factor model has been widely used in the processing and research of high-dimensional data and has achieved good results in constructing economic indicators and forecasting. One of the core theoretical ideas of the dynamic factor model assumes that the change of the high-dimensional time series set *X*_*t*_ can be driven by two parts. One part is driven by the intrinsic dynamic factor *f*_*t*_, and the other part is driven by the differential variation caused by the heterogeneous perturbation term *e*_*t*_ of the mean *О*. *e*_*t*_ contains the unique attribute or measurement error of a variable sequence at time *t*, and the change of dynamic factor *f*_*t*_ obeys a VAR process. The model of the dynamic factor can be expressed as follows:

(1) $$\begin{array}{c} X_{t}=\Lambda_{0}f_{t}+\Lambda_{1}f_{t-1}+...+\Lambda_{s}f_{t-s}+e_{t}. \end{array}$$

Here, *X*_*t*_ is an *N* × 1 time series observation variable, and *f*_*t*_ is a *q* × 1-dimensional potential dynamic factor, which affects all *N* sequences in *X*_*t*_. Λ_*j*_ is an *N* × *q*-dimensional dynamic factor loading matrix, *j* = 0,1,…,*s*, and *e*_*t*_ is a heterogeneous variable, which only affects a specific variable *X*_*t*_. The dynamic factor *f*_*t*_ obeys the VAR (*h*) process.

(2) $$\begin{array}{c} f_{t}=\Phi_{1}f_{t-1}+\Phi_{2}f_{t-2}+...+\Phi_{h}f_{t-h}+\varepsilon_{t}. \end{array}$$

Here, *ε*_*t*_ ~ *i*.*i*.*dN*(0, *Q*), and we assume that {*e*_*t*_}_1_^*T*^ and {*ε*_*t*_}_1_^*T*^ are independent of each other. It can be seen from the previous formula that the dynamics of the dynamic factor model are mainly reflected in two aspects. One, equation (1) contains a lag term, which accounts for the dynamics between the variable *X*_*t*_ and the factor *f*. Second, it can be seen from equation (2) that the dynamic factor itself obeys a VAR process, which reflects the dynamic nature of the factor. These two dynamics are the biggest difference from the static factor model. If *s* = 0, there is no lag term in equation (1), and the model becomes a static factor model. The usual factor analysis model does not take into account the dynamics between *X* and factor *f*. Therefore, for time series data, it is more reasonable to use a dynamic factor model for analysis. The dynamic factor model can be expressed in the form of the following static factors:

(3) $$\begin{array}{c} \Lambda =\left(\Lambda_{0},\Lambda_{1},...,\Lambda_{s}\right),\\ F_{t}={\left(f_{t}^{\prime },f_{t-1}^{\prime },...,f_{t-s}^{\prime }\right)}^{\prime }. \end{array}$$

We can get

(4) $$\begin{array}{c} X_{t}=\Lambda F_{t}+e_{t}. \end{array}$$

Here, the number of static factors is *q* × (*s* + 1), and the dimension of *F* is *q* × (*s* + 1).

The number of states of the Markov mechanism transition vector autoregressive model is *M*, and the observation time series *y* can be generated in the following ways:

(5) $$\begin{array}{c} P\left(y_{t}|Y_{t-1},s_{t}\right)=\left\{\begin{array}{c} f\left(y_{t}|Y_{t-1},\theta_{1}\right),s_{t}=1\\ \cdots \\ f\left(y_{t}|Y_{t-1},\theta_{M}\right),s_{t}=M. \end{array}\right. \end{array}$$

Here, *θ*_*m*_ is the parameter vector of the VAR model in the zone *m* = 1,2,…,*M*, and *Y*_*t*−1_ is the time series observation vector {*Y*_*t*−1_}_*j*=1_^*∞*^. By giving a state *s*_*t*_, the *P*-order vector autoregressive model a of *y*_*t*_ can be obtained.

(6) $$\begin{array}{c} y_{t}=v\left(s_{t}\right)+\sum_{j=1}^{P}A_{j}\left(s_{t}\right)y_{t-j}+u_{t}. \end{array}$$

Here, *u*_*t*_ is a white noise process with 0 mean and covariance matrix ∑(*s*_*t*_), that is, *u*_*t*_ ~ *N*(0, ∑(*s*_*t*_)), whose value is related to the unobservable state variable *s*. In the MS-VAR model, *s*_*t*_ is assumed to consist of a uniform Markov chain of discrete states: Pr(*s*_*t*_*|*{*s*_*t*−*j*_}_*j*=1_^*∞*^, {*y*_*t*−*j*_}_*j*=1_^*∞*^)=Pr(*s*_*t*_*|s*_*t*−*j*_, *ρ*). The state transition probability *P*_*ij*_=Pr(*s*_*t*_*|*{*s*_*t*+1_=*j|s*_*t*_=*i*}) is the probability of going from state *i* to state *j*. Under the premise that state *i* is determined, it has nothing to do with the previous state. The transition probability matrix is

(7) $$\begin{array}{c} \left[\begin{array}{cccc} P_{11} & P_{12} & \cdots & P_{1M}\\ P_{21} & P_{22} & \cdots & P_{2M}\\ \cdots & \cdots & \cdots & \cdots \\ P_{M1} & P_{M2} & \cdots & P_{MM} \end{array}\right]. \end{array}$$

Here, ∑_*j*=1_^*M*^*P*_*j*_, ∀*i*, *j* ∈ {1, ..., *M*}. If *u*_*t*_ ~ *N*(0, ∑(*s*_*t*_)), the equation can be expressed in the following mean-adjusted form:

(8) $$\begin{array}{c} y_{t}-\mu =\sum_{j=1}^{P}A_{j}\left(s_{t}\right)\left(y_{t-j}-\mu \right)+u_{t}. \end{array}$$

MS-VAR models are subdivided into different class models based on whether the mean, variance, intercept term, and regression coefficients depend on state transition variables.

The sentiment value of the topic needs to be obtained from the document. Since the documents can be arranged according to time, it can be seen from the analysis results of the above LDA that for any day *t*, *n*_*t*_^*a*^ represents all the articles on the *t*-th day. For any article *n* on day *t*, there is always a topic *k* with the highest degree of relevance to it. This paper uses *D*_*t*,*n*,*k*_^*a*^ to describe the degree of relevance between the article and the topic (that is, the probability of the chapter corresponding to topic *k*). At this time, by calculating the sentiment value *S*_*t*,*n*_^*a*^ of the article *n*^*a*^, according to the calculation formula,

(9) $$\begin{array}{c} S_{t,k}=S_{t,n^{a}}\times D_{t,n^{a},k}, \end{array}$$

the sentiment value of topic *k* on day *t* can be obtained. If topic *k* appears more than once on day *t*, the sentiment value of all documents corresponding to topic *k* in the day is summed up as the sentiment value of topic *k* on that day. At this time, the emotional value of topic *k* on day *t* will be affected by two factors, one is the number of times topic *k* appears that day, and the other is the emotional value of topic *k* itself. The documents are sorted by time, and finally, the sentiment value matrix *D*_0_ of *T*^*d*^ × *K* can be obtained, where *T*^*d*^ represents the number of days in the sample interval, *K* represents the number of topics, and the value in the matrix is the sentiment value of the topic *k* on that day. Since the real GDP growth rate is quarterly data, the daily sentiment values of the topic are summed quarterly here and the summed values are subjected to min-max normalization processing by the column to obtain the quarterly sentiment matrix *D*_1_.

The dynamic factor model is used to estimate the common factors that drive the change in the target topic. When estimating, the number *r* of the factors to be estimated should be determined first. This paper uses the information criterion to perform the analysis. This criterion comprehensively considers the benefits and costs of adding factors and finally selects the number of factors *r* that minimizes the IC value as the optimal number of factors. The specific theory is as follows:

(10) $$\begin{array}{c} IC\left(r\right)=\ln V_{r}\left(\hat{\Lambda },\hat{F}\right)+rg\left(N,T\right). \end{array}$$

Here,

(11) $$\begin{array}{c} V_{r}\left(\hat{\Lambda },\hat{F}\right)=\min_{\Lambda }\frac{1}{NT}\sum_{i=1}^{N}\sum_{i=1}^{T}{\left(X_{it}-\lambda_{i}^{r}{\hat{F}}_{t}^{k}\right)}^{2}, \end{array}$$

is the residual sum of squares when the number of factors is determined as *k*,

(12) $$\begin{array}{c} g\left(N,T\right)=\frac{\left(N+T\right)\ln\left(\min\left(N,T\right)\right)}{\left(NT\right)}, \end{array}$$

is the penalty term, *N* is the number of observable variables, where *N* = 7 in this paper, and *T* is the length of time. This article aggregates daily data into quarterly data, so *T* = 63.

The above-mentioned method is used to test the optimal number of factors of the target theme emotion variable, and the test results are shown in Table 1.

It can be seen from the above-mentioned estimation results that when it is 1, the IC value is the smallest, which is 6.6333. Therefore, this paper extracts a dynamic factor and uses the Bayesian model estimation method based on MCMC to estimate the dynamic factor model.

## 4. Agricultural Economic Risk Forecast Based on Data Mining Technology

**Figure 3:**
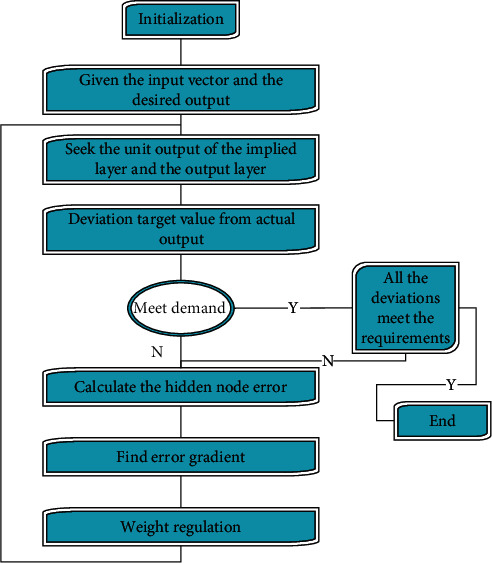
BP neural network algorithm flow.

This paper combines the BP neural network and the method of Section 3 to carry out agricultural economic data mining analysis. The risk assessment model of agricultural high-tech investment projects based on artificial neural networks is used to evaluate the risk of agricultural high-tech investment projects through the learning and training of neural networks and a series of evaluation indicators, strives to get rid of human subjective factors, and makes full use of the knowledge and experience of experts to provide support for relevant decision makers, as shown in Figure 3.

**Figure 4:**
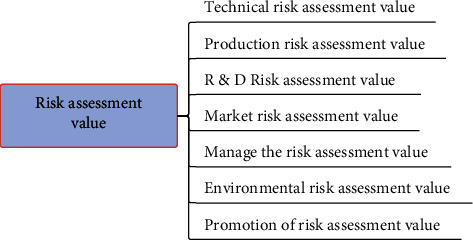
Risk forecast structure of the agricultural economy.

The risk assessment model of agricultural high-tech investment projects based on the BP artificial neural network in this paper is realized by the MATLAB6.0 program. Firstly, the risk assessment of the project is systematically modeled, and the structure of the risk assessment system model is shown in Figure 4.

Since there is no fixed risk evaluation reference value as a standard for the risk of agricultural high-tech investment projects, the establishment of this model is mainly used to select solutions from multiple projects. For a project, we can adjust the selection or carry out risk management on the invested project according to the size of the risk. When evaluating multiple projects, projects with low risks and high benefits are ideal choices, while projects with high risks and low benefits are generally not selected. When the risks and benefits of several projects are equal and it is difficult to select, the results are generally obtained by analyzing the size of “(1-risk)/benefits.” The smaller the result, the smaller the risk of the project's unit benefit.

Venture capital activities include four stages, namely, fund raising, fund investment, fund management, and fund exit. Fund raising stage refers to raising venture capital from various investors to form venture capital institutions or venture capital funds. The stage of capital investment means that after preliminary screening, value assessment, and due diligence of the start-up enterprises are qualified, the start-up enterprises are negotiated and signed relevant investment agreements, and finally, the venture capital is invested in the start-up enterprises in various ways. The fund management stage refers to the value-added management of the invested company by participating in the board of directors of the invested company, participating in strategic decision-making, and assisting the company in its follow-up financing. The capital withdrawal stage refers to the capital withdrawal through the listing, acquisition and merger, and liquidation of the invested company, and the investment income will be distributed to each investor of the invested project.

**Figure 5:**
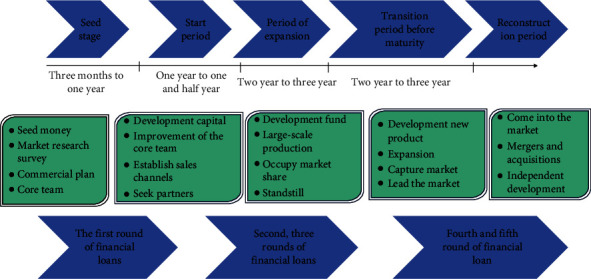
The process and stages of venture capital.

The process and stages of agricultural venture capital investment are shown in Figure 5.

**Figure 6:**
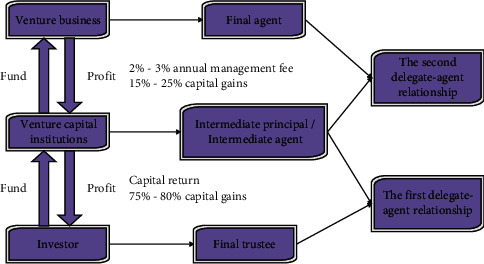
The dual principal-agent relationship and capital flow of agricultural venture capital.

Venture capital institutions are faced with a two-tier principal-agent relationship. During the operation of venture capital, the separation of capital ownership and control has been realized. The venture capital provider of the original capital provider forms the first-level principal-agent relationship with the venture capital institution as the venture capital manager. When a venture capital institution chooses a start-up enterprise for investment, a second-level principal-agent relationship is formed. The dual entrustment relationship of venture capital is shown in Figure 6.

**Figure 7:**
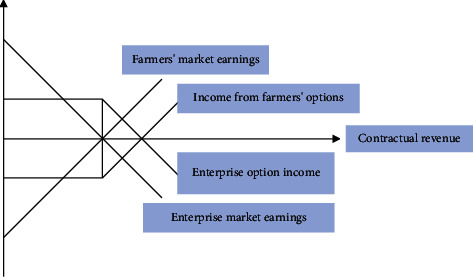
Income graph of the firm and farmers with fixed price.

Due to the limitations of contract farming itself, its role in risk avoidance is extremely limited. As a more advanced market form, the futures market can not only effectively avoid risks, but also provide a carrier for the operation of contract farming. Moreover, the two most important functions of futures trading are the risk transfer mechanism and price discovery mechanism. First, futures trading has a risk diversification and value preservation mechanism, which is conducive to the steady development of agricultural production. Spot trading cannot avoid the risk of loss due to market price fluctuations, while futures trading can make up for losses in the spot market, transfer risks through hedging, and reduce losses for producers and consumers. Therefore, when futures trading is used for hedging transactions in the opposite direction to spot trading, no economic loss will be caused no matter how the market conditions change. Therefore, the mutual offsetting of trading profits and losses in spot and futures transactions can reduce or avoid risks for producers and operators, so as to achieve the purpose of preserving value and obtaining better profits, which is conducive to the stable development of agricultural production. Secondly, the futures market has a mechanism to form “forward real prices,” which reduces the blindness and volatility of agricultural production in a market economy. The futures market price has strong anticipation and authenticity, can truly reflect the future market supply and demand situation in advance, and can also adjust the potential demand in future periods in advance. This avoids the blindness of producers' decision-making, reduces the large fluctuation of agricultural products, and ensures the basic balance of supply and demand of agricultural products in a certain period. Moreover, the expected income of producers has been guaranteed, the needs of consumers have also been met, and the stability and continuity of production and business activities have been maintained. Using the futures market to guide contract farming is the best model to transfer the risk of agricultural prices, and it is practically feasible. We take the contract revenue (market price) as the horizontal axis, and the vertical axis to represent the profit and loss of farmers or enterprises as shown in Figure 7.

**Figure 8:**
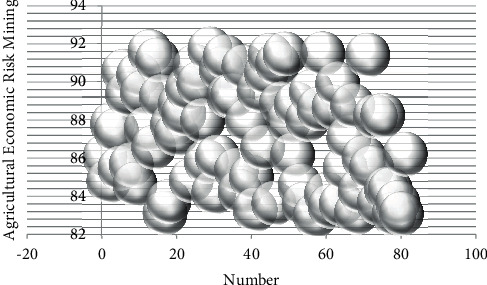
Bubble plot of the agricultural economic risk data mining.

**Figure 9:**
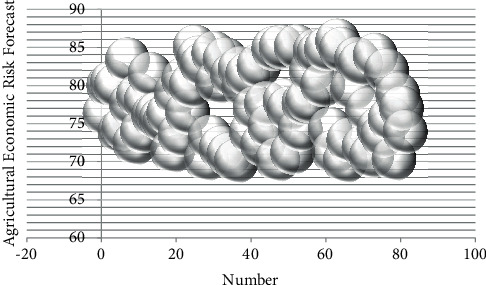
Bubble chart of agricultural economic risk factor forecast.

On the basis of the above-mentioned research, the effect of the agricultural economic risk forecast model based on data mining proposed in this paper is verified. In this paper, the simulation data is used to test, the agricultural economic data is obtained through the network, the data mining is carried out in combination with the model of this paper, and the agricultural economic risk is predicted. At the same time, this paper verifies the model proposed in this paper through simulation experiments, calculates the agricultural economic risk data mining effect and agricultural economic risk forecast effect of the system, and obtains the results shown in Tables 2 and 3 and Figures 8 and 9.

It can be seen from the above-mentioned research that the agricultural economic risk prediction system based on data mining technology proposed in this paper has a good effect.

## 5. Conclusion

In this paper, the BP neural network theory is used to combine the model theory with the data mining method. This realizes the characteristics of self-learning, self-adaptation, and generalization ability of the neural network and makes up for the uncertainty of the traditional early warning model which is difficult to deal with the highly nonlinear model. Moreover, it is technically feasible to successfully construct an agricultural financial risk early warning model based on the BP neural network. Similarly, the BP neural network technology can be used to construct macrorisk and microrisk early warning models for farmers, township enterprises, governments at all levels, agricultural financial regulatory agencies, and other agricultural financial entities. These models can guide agricultural financial institutions to take risk precontrol measures in advance to avoid losses caused by risks. In order to improve the ability to cope with agricultural economic risks, this paper studies the agricultural economic risk prediction combined with data mining technology and builds an intelligent agricultural economic risk prediction system. The simulation study shows that the agricultural economic risk prediction system based on data mining technology proposed in this paper has a good effect.

## Figures and Tables

**Table 1:** Test of the number of factors.

Number of factors	1	2	3	4	5	6	7
IC value	6.6333	6.8956	7.1682	7.4505	7.7411	8.0413	8.3441

**Table 2:** Statistical table of the agricultural economic risk data mining.

Number	Agricultural economic risk mining	Number	Agricultural economic risk mining	Number	Agricultural economic risk mining
1	86.08	28	85.83	55	88.04
2	84.82	29	91.71	56	89.47
3	87.78	30	84.23	57	83.02
4	87.45	31	86.08	58	88.59
5	85.49	32	90.65	59	91.46
6	90.55	33	91.30	60	84.13
7	89.35	34	89.26	61	83.50
8	85.67	35	89.27	62	88.67
9	84.65	36	85.38	63	89.89
10	90.37	37	84.27	64	83.63
11	89.43	38	90.78	65	85.49
12	87.54	39	87.98	66	87.08
13	91.56	40	85.02	67	88.75
14	86.57	41	83.32	68	83.16
15	91.14	42	89.57	69	84.33
16	89.22	43	86.57	70	86.04
17	83.14	44	90.48	71	91.39
18	83.75	45	91.38	72	85.50
19	87.77	46	83.65	73	88.19
20	87.28	47	90.96	74	83.65
21	88.81	48	88.85	75	88.24
22	88.33	49	91.45	76	84.38
23	89.66	50	87.95	77	84.36
24	84.85	51	86.18	78	83.08
25	90.80	52	88.82	79	83.69
26	89.90	53	84.57	80	83.11
27	87.97	54	83.61	81	86.22

**Table 3:** Forecast data of agricultural economic risk factors.

Number	Agricultural economic risk forecast	Number	Agricultural economic risk forecast	Number	Agricultural economic risk forecast
1	76.36	28	70.37	55	78.03
2	80.02	29	73.23	56	81.93
3	80.41	30	84.20	57	84.54
4	79.95	31	80.52	58	84.23
5	73.97	32	71.81	59	80.09
6	74.40	33	83.38	60	84.78
7	83.35	34	70.94	61	74.25
8	77.60	35	81.39	62	71.31
9	72.44	36	70.10	63	85.81
10	78.70	37	82.19	64	84.73
11	74.06	38	79.96	65	70.13
12	77.98	39	81.45	66	72.86
13	81.46	40	73.90	67	79.61
14	76.12	41	77.01	68	83.45
15	76.28	42	82.59	69	82.86
16	75.60	43	72.27	70	71.92
17	75.24	44	77.65	71	76.84
18	76.37	45	74.68	72	70.77
19	71.46	46	84.80	73	83.93
20	78.98	47	70.30	74	74.31
21	73.61	48	85.03	75	73.48
22	80.81	49	78.00	76	81.92
23	76.49	50	76.98	77	75.66
24	80.02	51	71.27	78	70.38
25	85.01	52	85.08	79	78.81
26	83.60	53	74.28	80	77.03
27	82.73	54	77.70	81	73.95

## Data Availability

The labeled datasets used to support the findings of this study are available from the corresponding author upon request.
